# Impaired postprandial GLP-2 response enhances endotoxemia, systemic inflammation, and kidney injury in metabolic dysfunction-associated steatohepatitis (MASH): effect of phospholipid curcumin meriva

**DOI:** 10.1080/19490976.2024.2424907

**Published:** 2024-12-02

**Authors:** Giovanni Musso, Mella Alberto, Filippo Mariano, Maurizio Cassader, Franco De Michieli, Antonella Riva, Giovanna Petrangolini, Stefano Togni, Silvia Pinach, Roberto Gambino

**Affiliations:** a MECAU Department San Luigi Gonzaga Hospital, Turin, Italy; b Department of Nephrology, Città della Salute e della Scienza Hospital, University of Turin, Turin, Italy; c Department of Medical Sciences, Città della Salute e della Scienza Hospital, University of Turin, Turin, Italy; d R & D Indena S.p.A, Milan, Italy

**Keywords:** Steatohepatitis, NF-kB, postprandial, eGFR, albuminuria, chemokine

## Abstract

We investigate the role of homeostatic mechanisms involved in acute, postprandial nutrient metabolism and nutrient-induced systemic inflammation in CKD presence and progression in Metabolic dysfunction-associated steatohepatitis (MASH). We assessed postprandial incretins (GLP-1 and GIP), intestinotropic hormone GLP-2, endotoxin LPS, Zonulin (a marker of intestinal permeability), hepatokines, adipokines and NF-kB activation in circulating MNCs during a meal tolerance test in 52 biopsy proven MASH patients randomized to curcumin Meriva or placebo and 26 matched controls. At baseline, MASH-CKD had a lower GLP-2 response and a 2-fold higher postprandial LPS and NF-kB activation in MNCs than MASH patients without CKD, but similar remaining postprandial or fasting parameters. Postprandial IAUC GLP-2 predicted the presence of CKD in MASH (OR = 0.43, 95%CI:0.32-0.80, *p* = 0.008) independently of liver histology and traditional risk factors. After 72 weeks, changes in IAUC GLP-2 independently predicted the presence of CKD (OR = 0.49, 95%CI:0.21-0.73, *p* = 0.010) and eGFR changes [β(SE) = 0.510(0.007, *p* = 0.006] at end-of-treatment, In MASH, an impaired GLP-2 response to meals is associated with intestinal barrier dysfunction, endotoxemia and NF-kB-mediated systemic inflammation and may promote renal dysfunction and CKD. These data provide the rationale for evaluating GLP-2 analogues in MASH-related CKD.

## Main text

Metabolic dysfunction-associated steatohepatitis (MASH), previously called Nonalcoholic steatohepatitis, NASH)^1,2^ is a multisystem disease with an increased risk of chronic kidney disease (CKD)^3–5^ and poor renal outcomes: consistently, MASH is the most rapidly growing indication for simultaneous liver-kidney transplantation.^6^ Mechanisms underlying kidney disease in MASH are unclear and none of the proposed MASH pharmacological treatments to date provides nephro-protection.^7^

MASH pathogenesis is multifactorial and involves genetic, epigenetic, nutritional, metabolic and inflammatory factors.^8–10^

The postprandial phase is the metabolic period during and after a meal (6–8 h), which involves the digestion and absorption of ingested nutrients and is characterized by an increase in glycemia, lipidemia and gut-derived endotoxemia, which promote systemic low-grade inflammation and are a pathogenic hallmark of metabolic syndrome.^11^ Consistently, the postprandial state is emerging as a key determinant of cardio-metabolic health.^11^

Although metabolic assessments are conventionally performed in the fasting state, in Western countries individuals spend most of the daytime in the postprandial phase: hypothesizing repetitive postprandial inflammatory bouts could promote CKD development and progression in MASH, we investigated

1)the link between postprandial nutrient homeostasis, nutritional inflammation and the development of CKD in MASH;

2)the effect of a novel, enhanced curcumin formulation on the above mechanisms and on kidney disease in MASH. Curcumin is a known stimulator of Glucagon-like Peptides secretion by enteroendocrine cells via different molecular mechanisms^12–14^

## Methods

We took advantage of a recent multicenter randomized trial assessing the effect of enhanced phospholipid curcumin formulation Meriva in MASH15: : in this trial, 52 biopsy-proven MASH patients were randomized to Meriva 2 g/d or placebo for 72 weeks, after stratification for baseline diabetes, liver fibrosis stage (F0–2 vs. F3–4), CKD (presence/absence), and obesity status. Chemical characteristics of curcumin Meriva formulation, trial design, methods and protocol and patient eligibility criteria are described in supplementary material and detailed elsewhere.^15^

All MASH patients underwent a standardized 8-hr oral mixed tolerance test at entry and at the end-of-treatment (EOT), with extensive metabolic and inflammatory assessment (Table 1), including measurement of circulating:

1. incretins (GIP and GLP-1) and GLP-2, a key intestinotrophic hormone released from enteroendocrine L cells;
2. zonulin, a marker of gut permeability, and lipopolysaccharide (LPS) a marker of endotoxemia,
3. hepatokine Fibroblast Growth Factor(FGF)-21;
4. adipokine adiponectin^16^;
5. the proinflammatory transcription factor Nuclear Factor (NF)-kB activation in blood MNCs and in the liver. NF-kB is a pivotal mediator of metabolic and nutritional inflammation, is activated by plasma glucose, non-esterified fatty acids (NEFAs) and LPS^17^ and inhibited by GLP-2.^18^

**Table 1.** Baseline and end-of-treatment (EOT) demographic, clinical, biochemical and histological characteristics of included MASH patients grouped according the the presence of chronic kidney disease (CKD)(*n* = 52) and 26 age-, gender-, BMI- and diabetes-matched controls.

	Controls (n = 26)	MASH CKD at baseline(n = 32)		MASH no CKD at baseline(n = 20)		P for between-group changes in
		Baseline	EOT	Baseline	EOT	MASH
MASH patients on active treatment n (%)	0(0%)	16(50%)	16(50%)	10(50%)	10(50%)	0.897
Demographics						
Age (years)	54 (12)	55 (11)	56 (11)	53 (10)	54 (10)	0.638
Male	13 (50%)	15 (47%)	15(47%)	11 (55%)	11 (55%)	0.489
Caucasian white race	52(100%)	30(94%)	30(94%)	19(95%)	19(95%)	0.528
Weight status						
Obesity Overweight	18(70%) 8 (30%)	23 (72%) 9 (28%)	23 (72%) 9 (28%)	14(70%) 6 (30%)	14 (70%) 6 (30%)	0.781 0.613
Comorbidities						
Type 2 diabetes	13(50%)	16(50%)	16 (50%)	9(45%)	9 (45%)	0.569
Hyperlipidaemia*	15 (58%)	16 (50%)	16(50%)	12 (60%)	12 (60%)	0.478
Hypertension†	19 (73%)	18 (56%)	18 (56%)	12(60%)	12 (60%)	0.396
Cardiovascular disease	0(0%)	1 (3%)	1 (3%)	1 (5%)	1 (5%)	0.791
Current smoker	5 (19%)	3 (9%)	3 (9%)	4 (20%)	4 (20%)	0.582
Medications						
Anti-diabetic						
Metformin	15(58%)	14 (44%)	14 (44%)	10 (50%)	10 (50%)	0.419
Sulfonylurea	1 (4%)	1 (3%)	1 (3%)	1 (5%)	1 (5%)	0.649
DPP-IV inhibitors	4 (9%)	3 (9%)	3 (9%)	2 (10%)	2 (10%)	0.82
Insulin	9(28%)	9(28%)	9 (28%)	6(30%)	7 (35%)	0.573
Anti-lipidaemic						
Statins PUFA Fibrates	10 (38%) 4 (15%) 1 (4%)	11 (34%) 3 (9%) 1 (3%)	11 (34%) 3 (9%) 1 (3%)	7 (35%) 2 (10%) 0 (0%)	7 (35%) 2 (10%) 0 (0%)	0.396 0.847 0.840
Anti-hypertensive						
ACEI/ARB	14 (54%)	16 (50%)	16 (50%)	11 (55%)	11 (55%)	0.893
Others	13 (50%)	15 (47%)	15 (47%)	11 (55%)	11 (55%)	0.528
Anti-platelet	3 (12%)	3 (9%)	3 (9%)	3 (15%)	3 (15%)	0.915
Genetic polymorphisms						
ApoE n(%)						
2-3 3-3 3-4	5(20) 16(60) 5(20)	7(22) 18(57) 7(21)	-	4(20) 11(55) 5(25)	-	0.895 0.724 0.812
PNPLA3 C/G n(%)						
C/C C/G G/G	15(58) 8(30) 3(12)	9(28) 14(44) 9(28)	-	5(25) 9(47) 6(28)	-	0.478 0.791 0.482
TM6SF2 C/T n(%)						
CC CT TT	22(85) 3(12) 1(3)	25(79) 5(15) 2(6)	-	14(72) 4(20) 2(8)	-	0.792 0.827 0.318
Dietary habits						
Total energy Intake (kcal/d)	2372 (169)	2501 (140)	2398 (151)	2569 (131)	2402 (128)	0.759
Kcal/kg BW/d	30 (1)	31 (1)	31 (1)	30 (1)	30 (1)	0.792
Alcohol (g/d)	4.0 (0.6)	4.2 (0.7)	4.0 (0.7)	4.0(0.9)	3.8 (0.8)	0.713
Fat (% kcal/d)	33.0 (0.9)	35.1 (0.8)	34.0 (0.9)	34.2 (0.9)	33.0 (1.0)	0.594
CHO (% kcal/d)	47.2 (1.8)	49.2 (1.1)	48.2 (1.1)	48.9(1.1)	46.4 (1.0)	0.573
Fiber (g/d)	28.9 (2.1)	26.5 (2.2)	27.9 (2.9)	24.9 (1.9)	25.2 (1.5)	0.692
Protein (% kcal/d)	18.5 (0.8)	16.1 (0.0)	14.8 (0.7)	15.1 (0.9)	14.2 (0.9)	0.429
SFA (% total fat)	33.6(0.8)	34.9 (0.4)	30.2 (0.4)	33.4 (0.9)	31.1 (0.7)	0.712
MUFA(% total fat)	46.6 (1.4)	47.8 (1.3)	48.9 (1.1)	46.2 (1.1)	47.2 (1.4)	0.639
PUFA(% total fat)	13.2 (1.1)	12.1 (0.8)	14.0 (0.9)	11.3 (0.9)	13.0 (1.2)	0.498
Physical activity (PA) categories						
Light PA (min/d)	169.9 (36.4)	171.8 (31.5)	171.8 (31.5)	193.4 (38.9)	193.4 (38.9)	0.813
Moderate PA (min/d)	102.6 (23.2)	98.6 (22.1)	101.6 (18.1)	99.7 (15.9)	103.4 (14.7)	0.849
Vigorous PA (min/d)	8.3 (8.2)	8.1 (7.3)	8.9 (6.3)	9.2 (7.2)	10.0 (9.1)	0.728
Sedentary time (min/d)	782.7 (88.7)	809.8 (80.4)	856.7 (91.2)	838.4 (91.3)	864.9 (89.9)	0.769
Metabolic parameters						
Weight (kg)	101.9 (9.7)	103.9 (15.9)	101.7 (19.4)	102.7 (18.5)	100.2 (15.3)	0.413
Body-mass index (kg/m ^2^	30.0(2.1)	33.4(3.1)	33.1 (2.7)	33.9 (3.2)	33.3 (2.9)	0.991
Waist circumference (cm)	95.3 (9.7)	107.3 (12.1)	104.7 (10.9)	106.1 (10.2)	105.1 (10.2)	0.739
HbA1c (%)	6.01 (0.47)	6.72 (0.51)	6.01 (0.43)†	6.68 (0.52)	6.03 (0.43)†	0.413
HbA1c (%) in T2DM	7.12 (0.79)	7.01 (0.52)	6.32 (0.44)†	7.12 (0.56)	6.25 (0.48)†	0.693
HOMA-IR	5.7 (2.4)	7.7 (4.9)	6.4 (3.2)†	7.9 (4.1)	6.1 (3.1)†	0.529
Total cholesterol (mg/dL)	175 (33)	196 (43)	167(38)†	199 (41)	162(37)†	0.492
LDL-C (mg/dL)	118 (21)	138 (24)	122 (18)†	136 (26)	119 (19)†	0.467
HDL-C (mg/dL)	43 (5)	39 (4)	46 (4)†	37 (4)	44 (4)†	0.518
oxLDL (IU/L)	46.7(11.2)	48.3 (17.5)	39.9 (19.9)	51.7 (21.8)	43.7 (19.7)	0.881
hsC-reactive protein (mg/L)	5.3 (3.8)	6.7 (4.1)	5.9 (3.1)	6.4 (3.8)	5.1 (3.9)	0.772
Systolic blood pressure (mm Hg)	127 (14)	130 (13)	127 (11)	133 (12)	130 (11)	0.594
Diastolic blood pressure (mm Hg)	76 (11)	79 (11)	78 (11)	78 (9)	75 (10)	0.395
Renal function						
Creatinine (mg/dL)	9.78 (0.20)	1.21 (0.20) ¶	0.89 (0.18)†	0.87 (0.17)	0.94 (0.28)†	0.128
eGFR (mL/min/1.73 m2)	96(4)	82(8) ¶	88(8)†	92(10)	97(10)†	0.216
eGFR stage (mL/min/1.73 m^2^)						
G1 (≥90 mL/min/1.73 m^2^)	26 (100%)	0 (0%)	13 (41%)†	20(100%)	16(80%)	0.009
G2(60-89)	0(0%)	22 (69%)	14 (44%)	0(42%)	3(15%)	0.169
G3a(45-59)	0(0%)	10 (31%)	5 (16%)	0 (0%)	1 (5%)	0.314
G3b (30-44)	0 (0%)	0 (0%)	0 (0%)	0 (0%)	0 (0%)	0.999
G4 (15-29)	0 (0%)	0 (0%)	0 (0%)	0 (0%)	0 (0%)	0.999
G5(<15)	0 (0%)	0 (0%)	0 (0%)	0 (0%)	0 (0%)	0.999
Albuminuria(AER)(mg/day)	43(25)	192(55) #	42(34)†	20(9)	61(22)	
A1 stage (<30 mg/day)	0(0%)	0(0%)	20(62%)†	20(100%)	16(80%)	0.160
A2 stage (30-300)	32(100%)	32(100%)	12(38%)†	0(0%)	4(20%)	0.211
A3 stage (>300)	0(0%)	0(0%)	0(0%)	0(0%)	0(0%)	0.999
CKD (eGFR stage≥G2 and/or albuminuria stage≥A2) n (%)	0 (0%)	32 (100%)#	12 (38%)‡	0(0%)	4(20%)	0.009
Liver function						
Alanine aminotransferase (U/L)	21 (8)	81 (28)	39 (22)†	73 (32)	37 (27)†	0.213
Aspartate aminotransferase (U/L)	17 (21)	57 (21)	26 (18)†	54 (24)	32 (20)*	0.393
γ-glutamyl transferase (U/L)	29 (18)	119 (68)	73 (61)*	112 (75)	72 (59)*	0.692
Alkaline phosphatase (U/L)	47 (21)	87 (25)	78 (29)	86 (38)	74 (39)	0.479
Total bilirubin (mg/dL)	0.82 (0.24)	0.82 (0.24)	0.73 (0.21)	0.70 (0.19)	0.60 (0.18)	0.394
Albumin (g/dL)	3.9 (0.7)	3.7 (0.6)	3.9 (0.6)	3.6(0.5)	4.0 (0.5)	0.529
Noninvasive markers of liver disease severity						
Comuterized sonographic Hepato/Renal ratio	0.92(0.31)	2.62(0.74)	1.12(0.47)†	2.60(0.60)	1.23(0.58)	0.397
Fatty liver Index	51(8)	99(12)	71(13	91(21)	79(18)	
Cytokeratin-18 fragments M30 (U/L)	101.4(22.4)	501.4(127.6) #	312.4(109.1)†	439.7(124.1)	297.2(112.1)†	0.284
FIB-4	1.01(0.21)	2.03(0.36)	1.57(0.39)*	1.97(0.33)	1.56(0.41)*	0.479
APRI	0.21(0.22)	0.71(0.23)	0.48(0.28)*	0.78(0.29)	0.51(0.20)*	0.528
Liver histology and Immmunohistochemistry						
Definite MASH	0(0%)	32 (100%)	21 (66%)	20 (100%)	11 (55%)	0.460
NAFLD activity score (0–8)	-	7.7 (0·8) ¶	4.2 (0·6)‡	5.2 (0·9)	3.6 (0·9)‡	0.129
Hepatocyte ballooning score (0–2)	-	1.7 (0.4)	1.3 (0.3)*	1.4 (0.5)	1.0 (0.3)*	0.314
Steatosis score (0–3)	-	2.2 (0.7)	1.2 (0.7)†	2.4(0.5)	1.3(0.5)†	0.485
Lobular inflammation score (0–3)	-	1.9 (0.6)	1.6 (0.5)†	1.5 (0.4)	1.1 (0.4)†	0.759
Kleiner fibrosis stage (0–4)	-	2.0 (1.3)	1.9 (1.3)	1.8 (0.9)	1.7(0.8)	0.748
Kleiner fibrosis stages						
F0 F1 F2 F3 F4	-	1 (3%) 6 (19%) 15(47%) 9 (28%) 2 (6%)	3 (9%) 11 (34%) 9(28%) 7 (22%) 3 (9%)	1 (5%) 7 (35%) 6(30%) 4 (25%) 1 (5%)	1 (5%) 6 (30%) 4(20%) 4 (20%) 2 (10%)	0.397 0.447 0.693 0.559 0.692
Clinically significant fibrosis (stage F2-4) n (%)	-	26 (81%) ¶	19 (73%)	11 (55%)	10 (50%)	0.394
Advanced fibrosis (stage 3-4) n (%)	-	11 (34%)	10 (31%)	5 (25%)	6 (30%)	0.893
Biopsy length (mm)	-	19.8 (5.1)	19.5 (5.4)	21.2 (4.6)	20.9 (4.9)	0.713
Number of portal tracts	-	19 (7)	20(7)	18 (6)	18 (6)	0.849
Hepatic nuclear NF-kB (% positive cells)	-	51(2)	27(2)‡	34(2)	21(2)‡	0.218
Standard Oral tolerance test						
Fasting Tg (mg/dL)	128 (27)	151 (33)	134 (35)	143 (29)	122 (24)	0.285
IAUC Tg (mg/dL x hr)	115(17)	197(21)	148(17)*	182(24)	132(21)*	0.297
Fasting NEFA (mmol/L)	0.31 (0.21)	0.56 (0.24)	0.46 (0.24)	0.52 (0.21)	0.41 (0.21)	0.313
IAUC NEFA (mMol/L x hr)	0.92(0.38)	1.97(0.43)	1.11(0.38)	1.81(0.48)	1.03(0.33)	0.339
Fasting LDL-C (mg/dL)	121 (16)	138 (24)	122 (18)*	136 (26)	119 (19)*	0.461
IAUC LDL-C (mg/dL x hr)	38(21)	43(22)	32(18)	48(24)	36(22)	0.398
Fasting plasma glucose (mg/dL)	104 (17)	118 (28)	110 (22)	117 (22)	109 (20)	0.397
Fasting GLP-1 (pmol/L)	6.9(2.2)	6.1(1.9)	7.1(2.9)	6.2(2.0)	6.9(1.9)	0.793
IAUC GLP-1 (pmol/L x hr)	17.4(3.9)	12.2(3.4)	17.1(3.2)†	12.9(3.1)	16.8(2.8)†	0.395
Fasting GLP-2 (ng/L)	11.9(1.5)	10.6(1.8)	11.1(2.0)	11.1(1.7)	11.6(1.9)	0.491
IAUC GLP-2 (ng/L x hr)	33.2(3.4)	12.5(2.1)#	29.1(2.7)†	17.9(2.4)	34.3(3.0)†	0.296
Fasting GIP (pg/mL)	10.6(1.2)	10.1(1.6)	10.9(1.5)	11.1(1.4)	10.7(1.9)	0.739
IAUC GIP (pg/mL x hr)	14.1(2.9)	12.1(3.1)	13.2(2,9)	13.4(2.8)	14.7(2.8)	0.513
fasting Zonulin (ng/mL)	465(82)	501(72)	486(78)	482(79)	469(78)	0.839
IAUC Zonulin (ng/mL x hr)	513(82)	1093(102)¶	607(81)	788(81)	496(73)	0.193
Fasting plasma LPS (IU/mL)	4.9(1.0)	5.1(1.0)	4.9(1.0)	4.7(0.9)	4.5(1.0)	0.392
IAUC plasma LPS (IU/mL x hr)	9.7(1.4)	47.2(3.9)#	10.7(1.2)†	29.9(3.4)	9.3(1.8)†	0.129
Fasting FGF-21 (pg/mL)	224(29)	427(41)	321(46)	397(37)	348(39)	0.397
IAUC FGF-21 (pg/mL x hr)	174(37)	38(19)	168(39)†	21(13)	171(36)†	0.491
Fasting adiponectin (ng(mL)	11032 ± 839§	6871 ± 610	7014 ± 570	6497 ± 404	6816 ± 611	0.779
IAUC adiponectin (ng/mL x hr)	9504 ± 918§	-1947 ± 510	5417 ± 371†	-2039 ± 521	5917 ± 438†	
Fasting MCP-1 (pg/mL)	163(28)	193(32)	179(24)	181(28(	175(22)	0.312
IAUC MCP-1 (pg/mL x hr)	139(31)	541(44) #	271(31)‡	377(39)	263(32)†	0.485
nuclear NF-κB in MNCs (%DNA binding activity)	26(2)	28(2)	26(2)	26(2)	25(2)	0.691
IAUC nuclear NF-κB in MNCs (%DNA binding activity x hr)	79(18)	316(48) #	76(13)‡	142(36)	58(11)‡	0.198

Data are presented as n(%) or mean ± SEM.

Abbreviations: Tg: triglyceride; C: cholesterol; FGF: Fibroblast Growth Factor; MASH: Metabolic dysfunction-associated steatohepatitis; MCP: monocyte chemoattractant protein; MNCs: mononuclear cells: NF-κB: nuclear factor-κB; HOMA-IR=homeostasis model assessment of insulin resistance. DPP-IV: dipeptidyl peptidase (DPP) IV inhibitors; oxLDL: oxidized LDL; Tg: triglyceride.

*.*p* < 0.05 vs. baseline within the same group (MASH-CKD or MASH without CKD).

†.*p* < 0.01 vs. baseline within the same group.

‡.*p* < 0.001 vs. baseline within the same group.

^¶^*p* < 0.01 vs. MASH without CKD at baseline.

^#^*p* < 0.001 vs. MASH without CKD at baseline.

§p < 0.05 vs MASH.

ԥ *p* < 0.01 vs MASH.

**Hyperlipidaemia** was defined as recorded in the past medical history, as receiving lipid-lowering drugs (eg, statin, fibrate, ezetimibe), or both.

**Hypertension** was defi ned as recorded in the past medical history, as receiving an anti – hypertensive drug, or both. ‡LDL concentration was calculated using the Friedewald formula.

**Non-alcoholic fatty liver disease (NAFLD) activity score** is the algebraic sum of steatosis score, lobular inflammation score and hepatocyte ballooning score.

**estimated Glomerular Filtration Rate (eGFR)** was assessed from serum creatinine using the CKD-EPI (Chronic Kidney Disease Epidemiology Collaboration) equation, as recommended by KDIGO guidelines. eGFR and urinary albumin excretion rate (AER) were classified according to Kidney Disease Improving Global Outcomes (KDIGO) categories.

**Chronic kidney disease (CKD)** was defined according to KDIGO guidelines as sustained (i.e., documented on 2 occasions at least 3 months apart) reduction in eGFR <60 mL/min/1.73 m2 and/or sustained increase in AER ≥30 mg/d. CKD regression was defined as sustained (i.e., documented 3 month apart, on both follow-up visit 7 and visit 8) eGFR ≥60 mL/min/1.73 m2 **and** normalization of AER (i.e., <30 mg/d).

HOMA-IR=homeostasis model assessment-estimated insulin resistance.

NF-kB activation in circulating mononuclear cells (MNCs) drives their differentiation into an inflammatory phenotype, recruits them to target organs and has been implicated in experimental models of obesity/overnutrition-induced MASH and CKD and in human diabetic nephropathy^19–21^

1. Monocyte Chemoattractant Protein (MCP)-1, a downstream NF-kB target gene and a pivotal chemokine promoting monocyte recruitment to target organs in MASH and CKD (supplementary material).

We also included 26 age-, gender, BMI-, diabetes-matched controls without liver or kidney disease, who underwent the same assessment as MASH patients, including the meal tolerance test.

Details on sample size estimation, controls selection and analytics are provided in supplementary material.

The following outcomes were assessed:

1. CKD presence and regression, defined according to Kidney Disease Improving Global Outcomes (KDIGO) guidelines as sustained (i.e., documented 3 months apart, on both follow-up visit 7 and visit 8) eGFR ≥90 mL/min/1.73 m^2^ (as assessed by CKD-EPI equation) **and** normalization of albumin excretion rate (AER)(i.e., <30 mg/d)(details in supplementary material).
2. change in absolute values and in KDIGO categories of eGFR and AER.

All data were analyzed on an intention-to-treat basis.

Subjects with and without CKD at EOT were compared for baseline values and for changes during follow-up. Normality was evaluated by Shapiro-Wilk test and non-normal values were log-transformed for regression analysis. Fisher or chi square test were used to compare categorical variables, as appropriate. Differences across groups were analyzed by ANOVA and then by Bonferroni correction, when variables were normally distributed; otherwise the Kruskal-Wallis test, followed by the post hoc Dunn test, was used to compare nonparametric variables.

To adjust for multiple comparison testing, the Benjamini-Hochberg False Discovery Rate correction was applied to raw p-values in all comparisons; significance was set at an adjusted p-value threshold of 0.05.

For the meal tolerance test, the area under the curve (AUC) and incremental AUC (IAUC) of parameters were computed by the trapezoid method and compared with ANOVA repeated measures.

Univariate and subsequent multivariate regression analyses were used to identify predictors of CKD at baseline and of CKD absence at EOT in MASH patients.

Adjustments was made for treatment allocation and for variables *a priori* known to be associated with CKD progression (age, baseline eGFR, baseline urinary AER, diabetes, baseline hepatic fibrosis stage (F0–2 vs F34), systolic blood pressure, LDL-cholesterol, HbA1c change, angiotensin-converting enzyme/angiotensin receptor blocker/statin use. Additional covariates were selected from parameters which differed at baseline between MASH patients with CKD and MASH without CKD. Continuous outcome measures were compared using linear regression, adjusting for parameter baseline values and allocated treatment (as model covariates, equivalent to ANCOVA), with multilevel modeling for key continuous outcome measures to account for repeated measures within each patient.

Use of GLP-1 analogues or SGLT2 inhibitors was an exclusion criterion from the trial.

## Results

At baseline, 32 (61%) out of 52 MASH patients had CKD and 20 (39%) had normal renal function: despite similar fasting biochemical, genetic, clinical and metabolic profile, after the meal challenge MASH patients with CKD showed impaired GLP-2 response, increased intestinal permeability and endotoxemia, and higher NF-kB activation in circulating MNCs than MASH patients without CKD, with LPS and NF-kB activation in MNCs peaking 2 hours after GLP-2 (Table 1, Figure 1a–c).

**Figure 1a.**
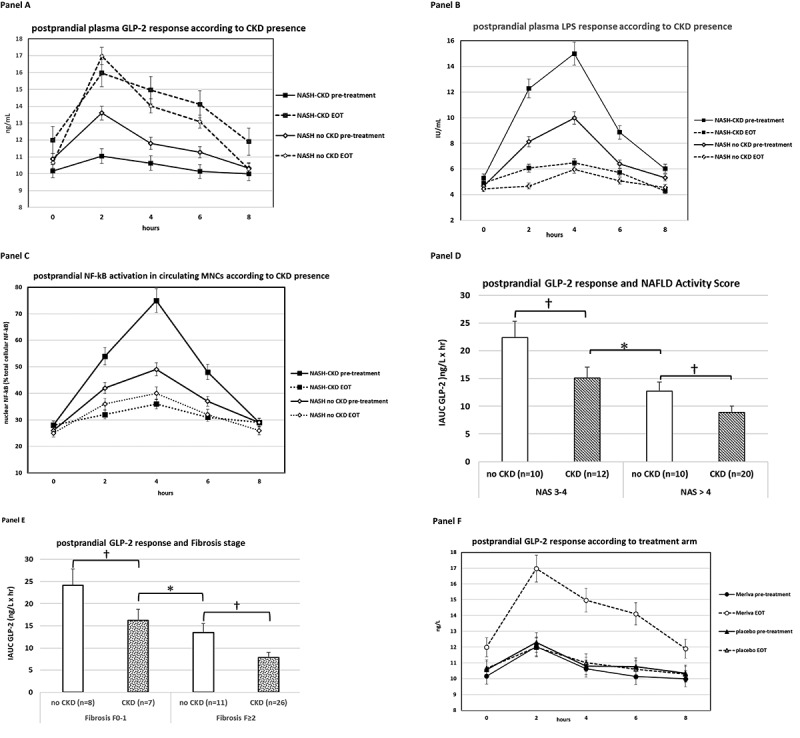
Panel A: GLP-2 response during the standardized meal tolerance test. Patients (*n* = 52) were grouped according to the presence (*n* = 32) or absence (*n* = 20) of chronic kidney disease(ckd) at baseline. Panel B: plasma lipopolysaccharide(lps) during the standardized meal tolerance test. Patients (*n* = 52) were grouped according to the presence (*n* = 32) or absence (*n* = 20) of chronic kidney disease(ckd) at baseline. Panel C: nf-kB activation in circulating mononuclear cells (MNCs) during the standardized meal tolerance test according to the presence (*n* = 32) or absence (*n* = 20) of chronic kidney disease(ckd) at baseline. Panel D: GLP-2 response during the standardized meal tolerance test at baseline. Patients (*n* = 52) were grouped according to liver disease activity, quantified as NAFLD activity Score(NAS). NAS is the algebraic sum of steatosis score (range 0-3), lobular inflammation (range 0-3) score and hepatocyte ballooning score (range 0-2). A NAS > 4 indicates high disease activity. Panel E: GLP-2 response during the standardized meal tolerance test at baseline. Patients (*n* = 52) were grouped according to fibrosis score (range 0-4). A fibrosis score ≥ 2 indicates clinically significant fibrosis. Panel F: GLP-2 response during the standardized meal tolerance test. Patients (*n* = 52) were grouped according to treatment allocation (phospholipid curcumin meriva or placebo). Panel G: changes (%) in nf-kB activation in circulating mononuclear cells (MNCs) during the standardized meal tolerance test. Patients (*n* = 52) were grouped according to treatment allocation (phospholipid curcumin meriva or placebo). Panel H: correlation between changes in postprandial GLP-2 response and changes in CKD status (present/absent) during the treatment in the whole NASH s population (*n* = 52). Panel I: correlation between changes in postprandial GLP-2 response and changes in absolute values of eGFR during the treatment in the whole NASH s population, grouped according to treatment arm (*n* = 52).

**Figure 1b.**
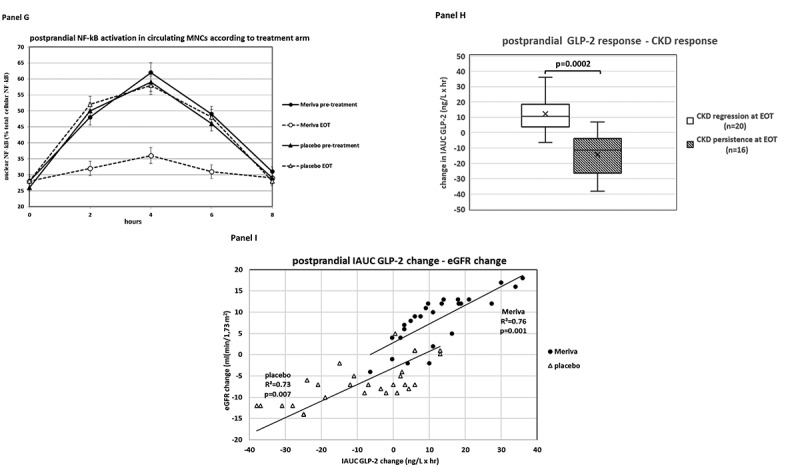
Continue

Consistent with existing literature^3,4^ NASH patients with CKD tended to have higher baseline histological NAFLD activity score (NAS) and a higher prevalence of clinically significant (stage F2–4) fibrosis than patients without CKD (Table 1). However, when grouping MASH patients according to the severity of histological disease activity (NAS ≤4 vs. NAS > 4) and of fibrosis (Fibrosis stage F0–1 vs. stage ≥F2), MASH patients with CKD showed an impaired postprandial GLP-2 response compared with patients without CKD (Figure 1d–e). Consistently, on multivariate regression analysis postprandial GLP-2 response was inversely associated with CKD independently of histological liver disease severity and of traditional CKD risk factors (Table 2 panel A),

**Table 2.** Logistic regression analysis in the whole MASH population(*n* = 52) for the presence of CKD at baseline and at end-of-treatment (EOT).

Panel A Outcome: presence of CKD at baseline		
Model	Odds ratio (95% CI)	p-value
Baseline hepatic fibrosis stage F2-4	1.79 (0.81-8.02)	0.106
Baseline NAFLD Activity Score (NAS)	1.48 (0.78-7.85)	0.382
Diabetes (present)	0.89 (0.48-4.32)	0.729
Obesity (present)	0.68 (0.10-6.79)	0.872
Dyslipidemia (present)	1.12 (0.39-12.73)	0.471
Hypertension (present)	0.52(0.14-4.91)	0.493
ACEIs/ARBs use	0.99 (0.80-4.37)	0.871
IAUC GLP-2 (highest tertile)	0.43 (0.32-0.80)	0.008
IAUC plasma LPS (highest tertile)	3.42 (0.89-7.75)	0.113
IAUC serum Zonulin (highest tertile)	1.94(0.39-3.71)	0.496
IAUC NF-kB activation in circulating MNCs (highest tertile)	3.29 (0.87-6.35)	0.109
IAUC plasma MCP-1 (highest tertile)	1.56 (0.39-3.18)	0.778
Panel B Outcome: CKD at EOT		
Model	Odds ratio (95% CI)	p-value
Age	0.99 (0.80-4.37)	0.871
Treatment allocation (curcumin)	4.99 (0.81-19.02)	0.112
HbA1c change	0.61 (0.18, 2.59)	0.692
eGFR at baseline	0.48 (0.09-4.83)	0.837
AER at baseline	0.26 (0.07-4.92)	0.328
Systolic BP change	0.73 (0.14-3.97)	0.512
LDL-cholesterol change	1.01 (0.39-4.26)	0.720
IAUC GLP-2 change	0.49 (0.21-0.73)	0.010
IAUC serum Zonulin change	0.98(0.24-4.94)	0.458
IAUC plasma LPS change	2.30(0.39-3.19)	0.315
IAUC NF-kB activation in circulating MNCs change(%)	3.31 (0.94-6.72)	0.111
IAUC plasma MCP-1 change	3.98(0.72-3.81)	0.112
Panel C Outcome: eGFR change at EOT		
Model	β (SE)	p-value
Age	-0.199 (0.127)	0.210
Treatment allocation (curcumin)	0.231(0.138)	0.227
HbA1c change	0.214(0.192)	0.613
eGFR at baseline	-0.332(0.003)	0.025
AER at baseline	-0.201(0.172)	0.308
Systolic BP change	-0.199(0.127)	0.210
LDL-cholesterol change	-0.288(0.139)	0.407
IAUC GLP-2 change	0.510(0.007)	0.006
IAUC serum Zonulin change	-0.284(0.009)	0.219
IAUC plasma LPS change	-0.391(0.198)	0.128
IAUC NF-kB activation in circulating MNCs change(%)	-0.310(0.012)	0.168
IAUC plasma MCP-1 change	-0.313(0.007)	0.068

All analyses will be carried out with Easy R ver1.61, Saitama, Japan. ^1^Kanda Y. Investigation of the freely available easy-to-use software ‘EZR’ for medical statistics. Bone Marrow Transplant. 2013;48:452-8.

Collectively, these findings point to an impaired postprandial GLP-2 response as a distinct feature of CKD in MASH.

Meriva treatment was associated with a significant increase in postprandial GLP-2 response, coupled with an improvement in postprandial intestinal permeability, endotoxemia and NF-kB activation in circulating MNCs (Table 1, Figure 1f–g).

At EOT, 20 (63%) of patients with baseline CKD showed regression of renal disease, while 4(20%) patients without CKD at baseline developed CKD (Table 1).

At follow-up data analysis, the changes in postprandial GLP-2 response significantly predicted the presence of CKD and eGFR changes at EOT on univariate and multivariate regression analyses, with the slope of correlation between changes in IAUC GLP-2 and in eGFR overlapping in the two treatment arms (Figure 1h–i, Table 2).

## Conclusion

In this study we scrutinized the major pathways involved in postprandial nutrient handling in biopsy-proven MASH patients with or without CKD: our findings disclosed for the first time an altered postprandial GLP-2/LPS/NF-kB pathway response as a distinct pathogenic feature of renal disease in MASH. Consistent with existing data,^4^ MASH patients with CKD tended to have more severe liver histology than MASH patients with normal renal function at baseline : by using univariate and multivariate analyses (Figure 1d–e, Table 2A) we dissected the effect of GLP-2 response to nutrients from that of baseline liver disease severity and of traditional risk factors for CKD and showed that impaired postprandial GLP-2 response was a distinct feature of renal disease in MASH. Baseline cross-sectional association between GLP-2 and CKD was confirmed longitudinally by exploring GLP-2/LPS/NF-kB pathway changes following treatment: in univariate analysis, the slope of correlation between changes in IAUC GLP-2 and in eGFR was statistically significant and similar between the two study arms, indicating a tight relationship between the two variables regardless of the type of treatment; more importantly, such relationship was confirmed in multivariate analyses, where changes in IAUC GLP-2 predicted eGFR changes and presence of CKD at EOT independently of treatment allocation, liver disease severity and known risk factors for CKD progression (Figure 1 panel I, Table 2). Collectively, these data suggest that CKD may signal dysfunctional GLP-2 response to meals in MASH, with repetitive, meal-associated bouts of endotoxemia and pro-inflammatory NF-kB activation in MNCs potentially injuring the kidney.

Thorough nutritional and metabolic assessment of MASH patients, who were also characterized in the postprandial phase, is a major strength of this report; the small sample size and the selected MASH population from two gastroenterological tertiary referral centers may however limit generalizability of our findings, which warrant confirmation in a larger unselected MASH population with the whole spectrum of CKD; furthermore, the relatively short duration of the follow-up, which was chosen following regulatory agencies’ recommendations for MASH drug development^22,23^prevented evaluation of hard liver-related and renal clinical outcomes.

If confirmed, the insights from this report may have therapeutically relevant implications: while previous reports suggested altered gut microbiota may contribute to CKD,^24^ identifying a specific downstream molecular pathway linking gut dysbiosis to systemic inflammation may be a more effective therapeutic strategy than gut microbiota manipulation with pro- or pre-biotics. There are several druggable targets in the GLP-2/LPS/NF-kB axis that are still unexplored in MASH,^25^ most prominently GLP-2 response. GLP-2 is an prominent regulator of intestinal permeability which is secreted by enteroendocrine L cells following meals and enhances intestinal barrier integrity: consistently, GLP-2 analogues^26^ are currently used in short bowel syndrome and may represent an attractive therapeutic tool in MASH by their anti-inflammatory effects, which are both indirect, mediated through amelioration of intestinal permeability and metabolic endotoxemia, and direct, by inhibition of NF-kB activation by LPS and NEFAs in MNCs.^7,26,27^

## Supplementary Material

NASH_NFkB_gut_microbes_R1_supplementary_methods clean.docx

## Data sharing statement

Deidentified participant data will be made available with publication to anyone upon reasonable request to corresponding author.
