# Supplementary material for: Impaired postprandial GLP-2 response enhances endotoxemia, systemic inflammation, and kidney injury in metabolic dysfunction-associated steatohepatitis (MASH): effect of phospholipid curcumin meriva
Source: Gut Microbes. 2024 Dec 2;16(1):2424907. doi: 10.1080/19490976.2024.2424907 (PMC11633829; doi:10.1080/19490976.2024.2424907)
Supplement: NASH_NFkB_gut_microbes_R1_supplementary_methods clean.docx [file KGMI_A_2424907_SM0019.docx]

**Supplementary material**

**Curcumin Meriva formulation.**  Curcumin Meriva® is a foodgrade formulation of curcumin in phospholipids that improves curcumin bioabsorption thanks to the lecithin phospholipids delivery system. This system is able to optimize the bioabsorption of the extract, with a physical and not pharmacological mechanism, preventing curcumin self-aggregation^[[1]](#endnote-1)^. Moreover, curcumin-phospatidylcholine complex Meriva showed a more efficient curcuminoids biotransformation by the human gut microbiota compared to unformulated curcumin, without altering the natural profile of curcuma metabolites^[[2]](#endnote-2)^.

**Chronic Kidney Disease(CKD) definition**

CKD was defined as sustained (i.e., documented on 2 occasions 3 month apart) reduction of eGFR (i.e. <90 mL/min/1.73 m2, assessed from serum creatinine using the CKD-EPI equation) and/or increased urinary albumin excretion rate (i.e., AER≥30 mg/d)]^[[3]](#endnote-3)^

CKD regression was defined as sustained (i.e., documented 3 month apart, on both follow-up visit 7 and visit 8) normalization of eGFR  (i.e. ≥90 mL/min/1.73 m2) and of urinary AER (i.e., <30 mg/d)].

eGFR was assessed from serum creatinine using the CKD-EPI (Chronic Kidney Disease Epidemiology Collaboration) equation, as recommended by KDIGO guidelines^[[4]](#endnote-4)^. eGFR and AER were classified according to KDIGO categories^3^

**Metabolic dysfunction-associated steatotic liver disease (MASLD) exclusion in controls**. The presence of MASLD in controls was excluded by **all** of these criteria: significant alcohol consumption (>20 g/d in males and >10 g/d in females, assessed by a validated questionnaire) or viral hepatitis markers, known liver disease, ultrasonographic bright liver, a quantitative computerized sonographic liver/kidney ratio>1.49 (a cut-off with 100% sensitivity for histological hepatic steatosis>5%)^[[5]](#endnote-5)^ using a standardized protocol^[[6]](#endnote-6)^ , liver enzyme elevation(including serum ALT>30 IU/L in men and >20 IU/L in women), a fatty liver index>60^[[7]](#endnote-7)^.

**Sample size calculation.** There are no data on postprandial incretin and endotoxin responsei in MASH with CKD. Based on previous data on postprandial1lipid and FGF21 responses in MASLD^[[8]](#endnote-8)^, ^[[9]](#endnote-9)^, ^[[10]](#endnote-10)^, assuming an effect size of ≥1.5(OR for CKD) across postprandial lipemia and FGF21 response quartiles and allowing for a 10% drop-out rate, at least 16 subjects were needed to detect a significant (p<0.05) difference in CKD prevalence across triglyceride and FGF21 responses in MASH with a power of 80%.

**Standardized oral tolerance test.**

Within 1 month from enrollment and 1 month from EOT liver biopsy, all patients underwent a standardized oral tolerance test^[[11]](#endnote-11)^ for a total energy content of 766 kcal. And the following composition: 75.3 g fat (55.6% saturated fatty acids, 29.6% monounsaturated fatty acids, 14.8% polyunsaturated fatty acids), 595 mg cholesterol, 9.8 g protein, 23 g carbohydrate. The meal was consumed over 5 minutes; subjects were kept fasting on the test morning, and strenuous activity will be forbidden. Participants were encouraged to avoid strenuous physical efforts and to follow their usual diet during the 24 hours preceding the test.

Blood samples were drawn at 0 (baseline), 2, 4, 6, and 8 hours. Plasma total cholesterol (Chol), triglyceride (Tg), free fatty acids (FFA), glucose, insulin were measured by automated enzymatic methods.

**Nuclear Factor (NF)-κB activation in circulating mononuclear cells (MNCs) during the oral tolerance test**

Blood samples were collected in tubes containing Na-EDTA as an anticoagulant; 3.5 mL of the anticoagulated blood sample was layered over 3.5 mL of the PMNL isolation medium (Robbins Scientific Corp, Sunnyvale, CA). Samples were centrifuged at 450 x *g* in a swing outrotor for 30 min at 22 °C. At the end of centrifugation, 2 bands separate out at the top of the red blood cell pellet. The top band consisted of mononuclear leukocytes (MNCs), whereas the bottom band consisted of polymorphumuclear leukocytes (PMNs). The MNC and PMN bands were harvested with a Pasteur pipette, repeatedly washed with Hank’s balanced salt solution, and reconstituted to a concentration of 4 x 10^5^ cells/mL in Hank’s balanced salt solution. This method yields> 95% pure PMN and MNC suspensions^[[12]](#endnote-12)^.

NF-kBp50/p65 transcription factor assay kit was purchased from Cayman Chemical (Ann Arbor MI, USA). The method detects specific transcription factor DNA binding activity in nuclear extracts and cell lysates. A specific double stranded DNA sequence containing the NF-kB response element is immobilized onto the bottom of wells of a 96 well-plate. NF-kB in nuclear or cytoplasmatic extract binds specifically to the NF-kB response element. NF-kBp50/65 is detected by addition of specific primary antibody directed against NF-kBp50/65. A secondary antibody conjugated to Horseradish peroxidase is added to provide a sensitive colorimetric readout at 450 nm^[[13]](#endnote-13)^.

**Nuclear Factor(NF)-κB activation in the liver by immunohistochemistry**

The ImmunoCruz® Staining System will be used in deparaffinized liver tissue according to the manufacturer’s instructions (Santa Cruz Biotechnology, Santa Cruz, CA). Briefly, slides will be soaked in 3% hydrogen peroxide for 5 min, washed, and incubated in serum blocking solution for 20 min. Specimens will be then incubated with primary antibodies for 2 h at 37◦ C. Tissue samples will be probed with mouse monoclonal antibodies reactive to NF-*κ*B p65 (Santa Cruz Biotechnology). After rinsing, specimens will be

incubated with biotinylated secondary antibody and a horseradish peroxidase-streptavidin complex, for 30 min each. Tissue samples will be then colorized with DAB substrate, counterstained, mounted, and examined. NF-*κ*B immunoreactivity will be expressed as the % of positive cells/high-power field (×400)^[[14]](#endnote-14)^ and will also be semiquantitatively evaluated using a 4-point scoring system (0—no staining;

1—positive staining in *<*30% of cells/high-power field; 2— positive staining in 30–70% of cells/high-power field; 3—positive staining in *>*70% of cells/high-power field).

**Other laboratory measurements**

Participants were genotyped for  *the single nucleotide polymorphisms (SNPs) rs738409 C>G in patatin-like phospholipase-3 (PNPLA3)*  and *Transmembrane 6 superfamily member 2 gene (TM6SF2) TM6SF2 rs58542926 C>T* variant, which affect liver disease severity in MASLD, and for for *apoE* genotype, a key regulator of postprandial lipid metabolism which has been previously linked to the risk of MASLD, with the real-time allele discrimination method, using TaqMan Allelic Discrimination Assay (Applied Biosystems, Foster city, CA). The TaqMan genotyping reaction was run on an 7300HT Fast Real-Time PCR (Applied Biosystem).

Urinary creatinine and albumin were assayed using Beckman-Coulter DxC700 clinical chemistry analyzers.

Serum Insulin (Mercodia, Uppsala, Sweden), nonesterified fatty acids (Zen-Bio, Research Triangle Park).

Commercial ELISA Kits (DRG International) was used for the quantitative determination of total GLP1 and GIP levels in serum, with both intra- and inter-assay coefficients of variation were <10%

Total GLP-2 content in plasma was measured using a GLP-2 ELISA kit (Phoenix Pharmaceuticals, Burlingame, CA) according to the manufacturer’s protocol.

Serum zonulin levels were determined by ELISA (human zonulin ELISA kit by MyBioSource, catalog
number: MBS749365). Both inter-assay and intra-assay CV were <10%.

LPS were measured in serum using a commercial ELISA kit (Cusabio, Wuhan, China).
The standards and samples were plated for 2 h at room temperature into a micro-plate precoated with the antibody specific for LPS. After incubation, samples were read at 450 nm. Values were expressed as pg/mL; intra-assay and inter-assay coefficients of variation were <10%.

Monocyte chemoattractant protein-1(MCP-1) and adiponectin were assessed using ELISA methods (Roche Modular system, Roche Ltd, Lewes, UK): intra-assay and inter-assay coefficients of variation were <10%.

Serum FGF21 was assayed using an enzyme-linked immunosorbent assay kit (R&D Systems, USA).

Data are expressed as mean ± standard error of the mean (SEM). Differences across groups were analyzed by analysis of variance (ANOVA) and then by Bonferroni's correction, when variables were normally distributed; otherwise, Kruskal-Wallis' test, followed by Dunn's post-hoc test, was used to compare nonparametric variables. Normality was evaluated by Shapiro-Wilk's test. Fisher's exact test or the chi-square test were used to compare categorical variables, as appropriate.

Area under the curve (AUC) and incremental AUC (iAUC) of parameters measured during the oral fat test and the OGTT were computed by the trapezoid method. Multivariate repeated-measures ANOVA was used to test the interaction between time and group during the oral fat load test. When a significant interaction was found between factors, differences across groups were analyzed by ANOVA followed by Bonferroni's correction, if variables were normally distributed; otherwise, Kruskal-Wallis' test was performed, followed by Dunn's post-hoc test, to compare nonparametric variables. Differences were considered statistically significant at *P* < 0.05.

Analysis of dietary, anthropometric, and metabolic parameters was made using Spearman's correlation test. When a relation was found on univariate analysis, multiple regression analyses were used to estimate the relationship between different variables after log transformation of skewed data.

All analyses will be carried out with Easy R ver1.61, Saitama, Japan^[[15]](#endnote-15)^.

1. Cuomo J, Appendino G, Dern AS, Schneider E, McKinnon TP, Brown MJ, Togni S, Dixon BM. Comparative absorption of a standardized curcuminoid mixture and its lecithin formulation. J Nat Prod. 2011;74: 664-9 [↑](#endnote-ref-1)
2. Bresciani L, Favari C, Calani L. Francinelli V, Riva A. Petrangolini G, Allegrini P, Mena P, Del Rio D. The effect of formulation of curcuminoids on their metabolism by human colonic microbiota. Molecules **2020**, 25, 940. [↑](#endnote-ref-2)
3. Lameire NH, Levin A, Kellum JA, Cheung M, Jadoul M, Winkelmayer WC, Stevens PE; Conference Participants. Harmonizing acute and chronic kidney disease definition and classification: report of a Kidney Disease: Improving Global Outcomes (KDIGO) Consensus Conference. Kidney Int. 2021;100: 516-526 [↑](#endnote-ref-3)
4. Levey AS, Stevens LA, Schmid CH, Zhang YL, Castro AF 3rd, Feldman HI, Kusek JW, Eggers P, Van Lente F, Greene T, et al. CKD-EPI (Chronic Kidney Disease Epidemiology Collaboration) : A new equation to estimate glomerular filtration rate. Ann Intern Med 2009; 150: 604–612. [↑](#endnote-ref-4)
5. Webb M, Yeshua H, Zelber-Sagi S, Santo E, Brazowski E, Halpern Z, Oren R. Diagnostic value of a computerized hepatorenal index for sonographic quantification of liver steatosis. AJR Am J Roentgenol. 2009 ;192: 909-14. [↑](#endnote-ref-5)
6. Xia MF, Yan HM, He WY, Li XM, Li CL, Yao XZ, Li RK, Zeng MS, Gao X. Standardized ultrasound hepatic/renal ratio and hepatic attenuation rate to quantify liver fat content: an improvement method. Obesity (Silver Spring). 2012;20:444-52. [↑](#endnote-ref-6)
7. ][Miyake T](http://www.ncbi.nlm.nih.gov.offcampus.dam.unito.it/pubmed?term=Miyake%20T%5BAuthor%5D&cauthor=true&cauthor_uid=22331365), [Kumagi T](http://www.ncbi.nlm.nih.gov.offcampus.dam.unito.it/pubmed?term=Kumagi%20T%5BAuthor%5D&cauthor=true&cauthor_uid=22331365), [Hirooka M](http://www.ncbi.nlm.nih.gov.offcampus.dam.unito.it/pubmed?term=Hirooka%20M%5BAuthor%5D&cauthor=true&cauthor_uid=22331365). Metabolic markers and ALT cutoff level for diagnosing nonalcoholic fatty liver disease: a community-based cross-sectional study. [J Gastroenterol.](file:///C:\Users\e755997\Downloads\gastroenterology) 2012; 47: 696-703. [↑](#endnote-ref-7)
8. Musso G, Cassader M, De Michieli F, Rosina F, Orlandi F, Gambino R. Nonalcoholic steatohepatitis versus steatosis: adipose tissue insulin resistance and dysfunctional response to fat ingestion predict liver injury and altered glucose and lipoprotein metabolism. Hepatology. 2012;56:933-42 [↑](#endnote-ref-8)
9. Grandt J, Jensen AH, Werge MP, Rashu EB, Møller A, Junker AE, Hobolth L, Mortensen C, Johansen CD, Vyberg M, et al. Postprandial dysfunction in fatty liver disease. Physiol Rep. 2023 Apr;11(8):e15653. [↑](#endnote-ref-9)
10. Li X, Zheng K, Liu L, Zhang T, Gu W, Hou X, Geng J, Song G. Relationship of postprandial fibroblast growth factor 21 with lipids, inflammation and metabolic dysfunction-associated fatty liver disease during oral fat tolerance test. Front Endocrinol (Lausanne). 2024;15:1343853. [↑](#endnote-ref-10)
11. Kolovou GD, Watts GF, Mikhailidis DP, Pérez-Martínez P, Mora S, Bilianou H, Panotopoulos G, Katsiki N, Ooi TC, Lopez-Miranda J, et al. Postprandial Hypertriglyceridaemia Revisited in the Era of Non-Fasting Lipid Profile Testing: A 2019 Expert Panel Statement, Main Text. Curr Vasc Pharmacol. 2019;17:498-514. [↑](#endnote-ref-11)
12. Ghanim H, Abuaysheh S, Sia CL. Increase in plasma endotoxin concentrations and the expression of Toll-like receptors and suppressor of cytokine signaling-3 in mononuclear cells following a high-fat high-carbohydrate meal: implications for insulin resistance. Diabetes Care 2009;32:2281–2287 [↑](#endnote-ref-12)
13. Deopurkar R, Ghanim H, Friedman J, Abuaysheh S, Sia CL, Mohanty P, Viswanathan P, Chaudhuri A, Dandona P. Differential effects of cream, glucose, and orange juice on inflammation, endotoxin, and the expression of Toll-like receptor-4 and suppressor of cytokine signaling-3. Diabetes Care. 2010 May;33(5):991-7. [↑](#endnote-ref-13)
14. Ribeiro PS, Cortez-Pinto H, Solá S, Castro RE, Ramalho RM, Baptista A, Moura MC, Camilo ME, Rodrigues CM. Hepatocyte apoptosis, expression of death receptors, and activation of NF-kappaB in the liver of nonalcoholic and alcoholic steatohepatitis patients. Am J Gastroenterol. 2004;99:1708-17. [↑](#endnote-ref-14)
15. Kanda Y. Investigation of the freely available easy-to-use software 'EZR' for medical statistics. Bone Marrow Transplant. 2013;48:452-8 [↑](#endnote-ref-15)
